# Tetra­kis(triphenyl­arsane-κ*As*)silver(I) trifluoro­acetate hemihydrate methanol hemisolvate

**DOI:** 10.1107/S1600536812045072

**Published:** 2012-11-28

**Authors:** Seik Weng Ng

**Affiliations:** a Department of Chemistry, University of Malaya, 50603 Kuala Lumpur, Malaysia; b Chemistry Department, King Abdulaziz University, PO Box 80203 Jeddah, Saudi Arabia

## Abstract

The Ag^I^ atom in the title hydrated solvated salt, [Ag(C_18_H_15_As)_4_](CF_3_CO_2_)·0.5CH_3_OH·0.5H_2_O, is coordinated by four As atoms from triphenyl­arsane ligands in a distorted tetra­hedral geometry. In the crystal, O—H⋯O hydrogen bonding occurs between carboxyl­ate groups of anions and lattice solvent mol­ecules. Of the four triphenyl­arsane ligands in the Ag complex cation, two each have an equally disordered phenyl ring while the trifluoro­acetate anion is disordered over two positions with respect to the lattice methanol and water mol­ecules which both show half-occupyncy. The crystal studied was a non-merohedral twin with a 13.6 (1)% contribution of the minor twin component.

## Related literature

For a related compound [Ag(C_18_H_15_P)_4_](CF_3_CO_2_)·C_2_H_5_OH, see: Ng (2012 ▶).
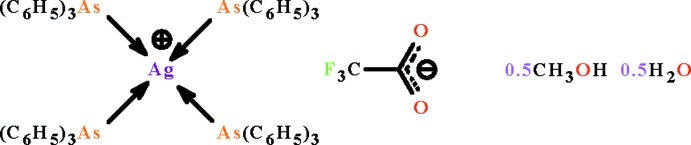


## Experimental

### 

#### Crystal data

[Ag(C_18_H_15_As)_4_](C_2_F_3_O_2_)·0.5CH_4_O·0.5H_2_O
*M* *_r_* = 1470.80
Triclinic, 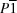

*a* = 11.9069 (2) Å
*b* = 14.5530 (3) Å
*c* = 18.5573 (4) Å
α = 88.893 (2)°
β = 85.782 (1)°
γ = 86.808 (1)°
*V* = 3201.57 (11) Å^3^
*Z* = 2
Mo *K*α radiation
μ = 2.42 mm^−1^
*T* = 100 K
0.22 × 0.17 × 0.11 mm

#### Data collection

Bruker SMART APEX diffractometer
Absorption correction: multi-scan (*TWINABS*; Bruker, 2009 ▶) *T*_min_ = 0.618, *T*_max_ = 0.776
48380 measured reflections
14449 independent reflections
10289 reflections with *I* > 2σ(*I*)
*R*_int_ = 0.058

#### Refinement

*R*[*F* ^2^ > 2σ(*F* ^2^)] = 0.048
*wR*(*F* ^2^) = 0.111
*S* = 1.03
14449 reflections
838 parameters
197 restraints
H-atom parameters constrained
Δρ_max_ = 1.08 e Å^−3^
Δρ_min_ = −0.56 e Å^−3^

### 

Data collection: *APEX2* (Bruker, 2009 ▶); cell refinement: *SAINT* (Bruker, 2009 ▶); data reduction: *SAINT*; program(s) used to solve structure: *SHELXS97* (Sheldrick, 2008 ▶); program(s) used to refine structure: *SHELXL97* (Sheldrick, 2008 ▶); molecular graphics: *X-SEED* (Barbour, 2001 ▶); software used to prepare material for publication: *publCIF* (Westrip, 2010 ▶).

## Supplementary Material

Crystal structure: contains datablock(s) global, I. DOI: 10.1107/S1600536812045072/xu5641sup1.cif

Structure factors: contains datablock(s) I. DOI: 10.1107/S1600536812045072/xu5641Isup2.hkl

Additional supplementary materials:  crystallographic information; 3D view; checkCIF report

## Figures and Tables

**Table 1:** Hydrogen-bond geometry (Å, °)

*D*—H⋯*A*	*D*—H	H⋯*A*	*D*⋯*A*	*D*—H⋯*A*
O3—H3⋯O1	0.84	1.97	2.80 (2)	175
O1w’—H1w1⋯O1′	0.84	2.02	2.86 (3)	179

**Fig. 1.**
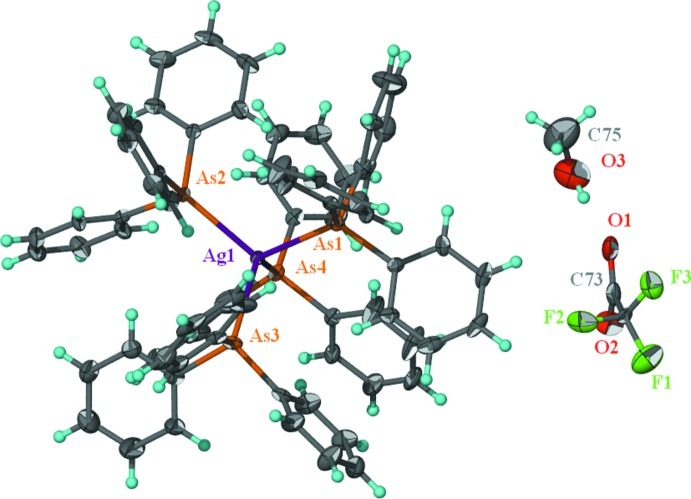
Thermal ellipsoid plot (Barbour, 2001) of [Ag(C18H15As)4](CF3CO2).0.5H2O.0.5CH3OH at the 70% probability level; hydrogen atoms are drawn as spheres of arbitrary radius. The disorder is not shown, and only selected atoms are labeled owing to crowding.

**Fig. 2.**
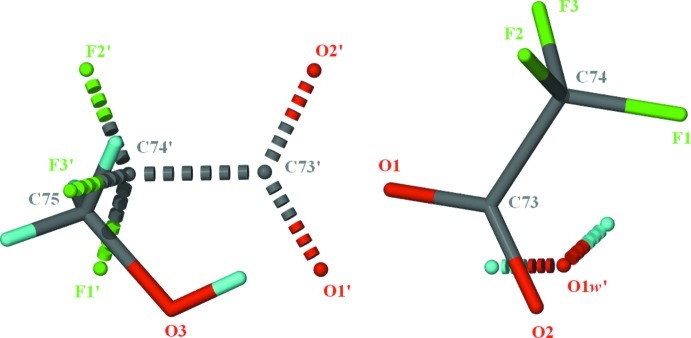
Disorder in the anion/solvent.

## supplementary crystallographic information

### Comment

Silver trifluoroacetate reacts with triphenylphosphine in a 1:4 stoichiometry in ethanol to from the salt, [Ag(C_18_H_15_P)_4_](CF_3_CO_2_)^.^C_2_H_5_OH (Ng, 2012). The corresponding reaction with triphenylarsane in methanol medium yielded [Ag(C_18_H_15_As)_4_](CF_3_CO_2_)^.^0.5H_2_O^.^0.5CH_3_OH, which has somewhat different solvent molecules (Scheme I, Fig. 1). Four arsane ligands bind to the metal atom; the trifluoacetate anion does not engaged in coordination, and is instead disordered with respect to methanol and water molecules. The disorder is such that the anion occupies two positions, as does the two solvent molecules (Fig. 2). The anion and solvent are engaged in weak hydrogen bonding (Table 1).

### Experimental

Silver trifluoroacetate (1 mmol) and triphenylarsane (4 mmol) were heated in methanol (25 ml) until the reactants dissolved completely. Colorless crystals were isolated from the filtered solution after several days.

### Refinement

Carbon-bound H-atoms were placed in calculated positions (C—H 0.95 to 0.98 Å) and were included in the refinement in the riding model approximation, with *U*(H) set to 1.2 to 1.5*U*(C).

The trifluoroacetate ion is disordered over two positions; as the occupancy refined to nearly 50%, the disorder was then fixed as a 1:1 type of disorder. The C–O distances were restrained to 1.25±0.01 Å, the C–C distances to 1.50±0.01 Å and the C–F distances to 1.35±0.01 Å. Additionally, the O···O distances were restrained to 2.17±0.01 Å and the F···F distances to 2.21±0.01 Å.

One half-occupancy trifluoroacetate portion is hydrogen bonded to a half-occupancy water molecule whereas the other half-occupancy portion is hydrogen bonded to a half-occupancy methanol molecule. For the methanol molecule, the C–O distance was restrained to 1.45±0.01 Å. The H atoms were placed in calculated positions (O–H 0.84 Å) and their temperature factors tied by a factor of 1.5 times.

The anisotropic temperature factors of the disordered atoms were restrained to be nearly isotropic.

Of the four triphenylarsane groups, two have each a disordered phenyl ring. The disorder was also treated as a 1:1 type of disorder. Pairs of As—C distances were restrained to within 0.01 Å of each other. The aromatic rings were refined as a rigid hexagon of 1.39 Å sides, and the temperature factors of the primed atoms were set to those of the unprimed ones. The anisotropic temperature factors were also restrained to be nearly isotropic.

The (1 0 0), (0 1 0) and (0 - 1 1) reflections that were affected by the beamstop as well as (-1 4 12) and (-3 8 1) were omitted.

The final difference Fourier map was featureless except for a peak at 0.88 Å from C74.

### Crystal data

**Table:**

[Ag(C_18_H_15_As)_4_](C_2_F_3_O_2_)·0.5CH_4_O·0.5H_2_O	*Z* = 2
*M**_r_* = 1470.80	*F*(000) = 1480
Triclinic, *P*1	*D*_x_ = 1.526 Mg m^−^^3^
Hall symbol: -P 1	Mo *K*α radiation, λ = 0.71073 Å
*a* = 11.9069 (2) Å	Cell parameters from 4258 reflections
*b* = 14.5530 (3) Å	θ = 2.2–25.5°
*c* = 18.5573 (4) Å	µ = 2.42 mm^−^^1^
α = 88.893 (2)°	*T* = 100 K
β = 85.782 (1)°	Prism, colorless
γ = 86.808 (1)°	0.22 × 0.17 × 0.11 mm
*V* = 3201.57 (11) Å^3^	

### Data collection

**Table:**

Bruker SMART APEX diffractometer	14449 independent reflections
Radiation source: fine-focus sealed tube	10289 reflections with *I* > 2σ(*I*)
Graphite monochromator	*R*_int_ = 0.058
ω scans	θ_max_ = 27.5°, θ_min_ = 1.1°
Absorption correction: multi-scan (TWINABS; Bruker, 2009)	*h* = −15→15
*T*_min_ = 0.618, *T*_max_ = 0.776	*k* = −18→18
48380 measured reflections	*l* = 0→24

### Refinement

**Table:**

Refinement on *F*^2^	Primary atom site location: structure-invariant direct methods
Least-squares matrix: full	Secondary atom site location: difference Fourier map
*R*[*F*^2^ > 2σ(*F*^2^)] = 0.048	Hydrogen site location: inferred from neighbouring sites
*wR*(*F*^2^) = 0.111	H-atom parameters constrained
*S* = 1.03	*w* = 1/[σ^2^(*F*_o_^2^) + (0.0263*P*)^2^ + 7.5548*P*] where *P* = (*F*_o_^2^ + 2*F*_c_^2^)/3
14449 reflections	(Δ/σ)_max_ = 0.001
838 parameters	Δρ_max_ = 1.08 e Å^−^^3^
197 restraints	Δρ_min_ = −0.56 e Å^−^^3^

### Fractional atomic coordinates and isotropic or equivalent isotropic displacement parameters (Å^2^)

**Table:**

	*x*	*y*	*z*	*U*_iso_*/*U*_eq_	Occ. (<1)
Ag1	0.49421 (3)	0.25589 (3)	0.24626 (2)	0.01910 (9)	
As1	0.56948 (4)	0.42096 (3)	0.21599 (3)	0.02081 (12)	
As2	0.33767 (4)	0.21756 (3)	0.15901 (3)	0.01929 (12)	
As3	0.40995 (5)	0.25168 (4)	0.38150 (3)	0.02716 (13)	
As4	0.66244 (4)	0.12921 (4)	0.23054 (3)	0.02178 (12)	
C1	0.6503 (8)	0.4230 (7)	0.1228 (3)	0.0268 (14)	0.50
C2	0.7331 (8)	0.3536 (7)	0.1076 (5)	0.024 (2)	0.50
H2	0.7507	0.3091	0.1438	0.029*	0.50
C3	0.7901 (7)	0.3493 (6)	0.0396 (6)	0.049 (3)	0.50
H3A	0.8468	0.3018	0.0292	0.059*	0.50
C4	0.7643 (8)	0.4144 (7)	−0.0133 (4)	0.051 (3)	0.50
H4	0.8033	0.4114	−0.0599	0.061*	0.50
C5	0.6815 (9)	0.4838 (6)	0.0018 (4)	0.062 (3)	0.50
H5	0.6639	0.5283	−0.0344	0.075*	0.50
C6	0.6245 (7)	0.4881 (6)	0.0698 (5)	0.036 (2)	0.50
H6	0.5678	0.5356	0.0802	0.043*	0.50
C1'	0.6480 (8)	0.4370 (7)	0.1207 (3)	0.0268 (14)	0.50
C2'	0.7066 (9)	0.3600 (6)	0.0906 (5)	0.024 (2)	0.50
H2'	0.6996	0.3014	0.1134	0.029*	0.50
C3'	0.7754 (8)	0.3689 (6)	0.0273 (6)	0.049 (3)	0.50
H3'	0.8154	0.3162	0.0068	0.059*	0.50
C4'	0.7856 (8)	0.4547 (7)	−0.0060 (4)	0.051 (3)	0.50
H4'	0.8326	0.4607	−0.0492	0.061*	0.50
C5'	0.7270 (8)	0.5317 (6)	0.0241 (4)	0.062 (3)	0.50
H5'	0.7340	0.5903	0.0013	0.075*	0.50
C6'	0.6582 (7)	0.5228 (6)	0.0874 (4)	0.036 (2)	0.50
H6'	0.6182	0.5754	0.1079	0.043*	0.50
C7	0.6762 (4)	0.4713 (3)	0.2772 (3)	0.0218 (11)	
C8	0.6637 (5)	0.4545 (4)	0.3509 (3)	0.0281 (12)	
H8	0.6078	0.4150	0.3703	0.034*	
C9	0.7330 (5)	0.4955 (4)	0.3966 (3)	0.0407 (15)	
H9	0.7244	0.4839	0.4472	0.049*	
C10	0.8142 (5)	0.5529 (4)	0.3686 (3)	0.0391 (15)	
H10	0.8601	0.5822	0.4001	0.047*	
C11	0.8287 (5)	0.5677 (4)	0.2952 (4)	0.0385 (15)	
H11	0.8864	0.6056	0.2758	0.046*	
C12	0.7594 (4)	0.5276 (4)	0.2496 (3)	0.0292 (12)	
H12	0.7691	0.5388	0.1990	0.035*	
C13	0.4587 (4)	0.5245 (3)	0.2146 (3)	0.0232 (11)	
C14	0.4769 (5)	0.6085 (4)	0.2426 (3)	0.0294 (12)	
H14	0.5480	0.6191	0.2601	0.035*	
C15	0.3922 (5)	0.6783 (4)	0.2456 (3)	0.0339 (13)	
H15	0.4055	0.7359	0.2655	0.041*	
C16	0.2898 (5)	0.6643 (4)	0.2199 (3)	0.0339 (14)	
H16	0.2324	0.7123	0.2213	0.041*	
C17	0.2709 (6)	0.5806 (5)	0.1920 (4)	0.055 (2)	
H17	0.1992	0.5700	0.1753	0.066*	
C18	0.3557 (5)	0.5109 (4)	0.1881 (4)	0.0480 (18)	
H18	0.3428	0.4539	0.1670	0.058*	
C19	0.2016 (6)	0.2973 (6)	0.1538 (7)	0.0200 (11)	0.50
C20	0.1681 (9)	0.3572 (7)	0.2097 (6)	0.032 (3)	0.50
H20	0.2105	0.3582	0.2511	0.039*	0.50
C21	0.0725 (10)	0.4158 (7)	0.2051 (6)	0.037 (3)	0.50
H21	0.0496	0.4568	0.2433	0.044*	0.50
C22	0.0105 (7)	0.4144 (7)	0.1446 (7)	0.0364 (17)	0.50
H22	−0.0549	0.4544	0.1415	0.044*	0.50
C23	0.0439 (7)	0.3544 (6)	0.0887 (6)	0.035 (3)	0.50
H23	0.0015	0.3535	0.0474	0.042*	0.50
C24	0.1395 (7)	0.2959 (5)	0.0933 (6)	0.029 (2)	0.50
H24	0.1624	0.2549	0.0551	0.034*	0.50
C19'	0.2035 (6)	0.2995 (6)	0.1534 (7)	0.0200 (11)	0.50
C20'	0.1488 (10)	0.3303 (7)	0.2179 (6)	0.032 (3)	0.50
H20B	0.1749	0.3089	0.2627	0.039*	0.50
C21'	0.0558 (10)	0.3923 (8)	0.2168 (6)	0.037 (3)	0.50
H21B	0.0184	0.4134	0.2609	0.044*	0.50
C22'	0.0176 (7)	0.4236 (7)	0.1512 (7)	0.0364 (17)	0.50
H22B	−0.0459	0.4660	0.1505	0.044*	0.50
C23'	0.0724 (8)	0.3928 (6)	0.0867 (6)	0.035 (3)	0.50
H23B	0.0463	0.4141	0.0419	0.042*	0.50
C24'	0.1653 (7)	0.3307 (6)	0.0878 (6)	0.029 (2)	0.50
H24B	0.2028	0.3097	0.0437	0.034*	0.50
C25	0.2712 (4)	0.0995 (3)	0.1782 (3)	0.0224 (11)	
C26	0.3435 (4)	0.0212 (4)	0.1775 (3)	0.0286 (12)	
H26	0.4221	0.0263	0.1667	0.034*	
C27	0.3007 (5)	−0.0643 (4)	0.1927 (3)	0.0363 (14)	
H27	0.3502	−0.1179	0.1918	0.044*	
C28	0.1862 (5)	−0.0720 (4)	0.2091 (3)	0.0326 (13)	
H28	0.1569	−0.1304	0.2200	0.039*	
C29	0.1156 (5)	0.0059 (4)	0.2094 (3)	0.0369 (14)	
H29	0.0369	0.0006	0.2198	0.044*	
C30	0.1568 (4)	0.0921 (4)	0.1948 (3)	0.0283 (12)	
H30	0.1071	0.1455	0.1961	0.034*	
C31	0.3875 (4)	0.2082 (3)	0.0575 (3)	0.0208 (11)	
C32	0.3434 (5)	0.1457 (4)	0.0127 (3)	0.0311 (13)	
H32	0.2907	0.1036	0.0324	0.037*	
C33	0.3763 (6)	0.1448 (4)	−0.0608 (3)	0.0424 (16)	
H33	0.3463	0.1020	−0.0912	0.051*	
C34	0.4523 (6)	0.2060 (4)	−0.0894 (3)	0.0411 (16)	
H34	0.4739	0.2058	−0.1398	0.049*	
C35	0.4975 (5)	0.2677 (4)	−0.0452 (3)	0.0387 (15)	
H35	0.5504	0.3094	−0.0652	0.046*	
C36	0.4658 (4)	0.2687 (4)	0.0281 (3)	0.0312 (13)	
H36	0.4975	0.3107	0.0583	0.037*	
C37	0.3449 (5)	0.1394 (4)	0.4188 (3)	0.0340 (13)	
C38	0.2712 (5)	0.0977 (4)	0.3775 (3)	0.0366 (14)	
H38	0.2553	0.1232	0.3316	0.044*	
C39	0.2206 (6)	0.0196 (5)	0.4024 (4)	0.0476 (17)	
H39	0.1690	−0.0083	0.3741	0.057*	
C40	0.2444 (7)	−0.0185 (6)	0.4683 (4)	0.072 (3)	
H40	0.2106	−0.0730	0.4856	0.086*	
C41	0.3192 (8)	0.0245 (6)	0.5094 (5)	0.081 (3)	
H41	0.3347	−0.0007	0.5555	0.098*	
C42	0.3707 (6)	0.1018 (5)	0.4851 (4)	0.056 (2)	
H42	0.4231	0.1292	0.5131	0.067*	
C43	0.5097 (5)	0.2796 (4)	0.4557 (3)	0.0286 (12)	
C44	0.6176 (5)	0.2395 (5)	0.4512 (3)	0.0444 (16)	
H44	0.6410	0.1976	0.4138	0.053*	
C45	0.6922 (6)	0.2611 (5)	0.5024 (3)	0.0473 (17)	
H45	0.7666	0.2337	0.4995	0.057*	
C46	0.6592 (5)	0.3214 (5)	0.5567 (4)	0.0441 (16)	
H46	0.7100	0.3347	0.5918	0.053*	
C47	0.5533 (6)	0.3620 (5)	0.5601 (4)	0.0517 (18)	
H47	0.5307	0.4047	0.5972	0.062*	
C48	0.4776 (5)	0.3412 (4)	0.5096 (3)	0.0406 (15)	
H48	0.4036	0.3697	0.5125	0.049*	
C49	0.2838 (5)	0.3400 (4)	0.4013 (3)	0.0303 (13)	
C50	0.1901 (5)	0.3177 (4)	0.4462 (3)	0.0372 (14)	
H50	0.1888	0.2595	0.4703	0.045*	
C51	0.0992 (5)	0.3804 (5)	0.4558 (4)	0.0477 (17)	
H51	0.0352	0.3654	0.4865	0.057*	
C52	0.1010 (6)	0.4646 (5)	0.4210 (3)	0.051 (2)	
H52	0.0380	0.5074	0.4275	0.061*	
C53	0.1939 (6)	0.4875 (5)	0.3765 (3)	0.0486 (18)	
H53	0.1947	0.5458	0.3526	0.058*	
C54	0.2865 (6)	0.4246 (4)	0.3669 (3)	0.0403 (15)	
H54	0.3509	0.4402	0.3368	0.048*	
C55	0.7410 (4)	0.1122 (4)	0.1358 (3)	0.0250 (11)	
C56	0.6771 (5)	0.1027 (4)	0.0766 (3)	0.0302 (13)	
H56	0.5972	0.1033	0.0834	0.036*	
C57	0.7298 (5)	0.0923 (4)	0.0079 (3)	0.0389 (15)	
H57	0.6855	0.0872	−0.0323	0.047*	
C58	0.8449 (6)	0.0894 (4)	−0.0029 (3)	0.0444 (16)	
H58	0.8804	0.0809	−0.0500	0.053*	
C59	0.9097 (5)	0.0989 (5)	0.0559 (3)	0.0486 (18)	
H59	0.9896	0.0973	0.0488	0.058*	
C60	0.8584 (5)	0.1105 (5)	0.1241 (3)	0.0403 (15)	
H60	0.9032	0.1176	0.1637	0.048*	
C61	0.7870 (4)	0.1530 (4)	0.2891 (3)	0.0253 (11)	
C62	0.8187 (5)	0.2417 (4)	0.2932 (4)	0.054 (2)	
H62	0.7826	0.2894	0.2662	0.065*	
C63	0.9038 (6)	0.2618 (5)	0.3370 (5)	0.064 (2)	
H63	0.9235	0.3238	0.3411	0.077*	
C64	0.9581 (5)	0.1952 (5)	0.3733 (3)	0.0463 (17)	
H64	1.0181	0.2095	0.4015	0.056*	
C65	0.9280 (5)	0.1078 (6)	0.3700 (4)	0.058 (2)	
H65	0.9659	0.0608	0.3967	0.070*	
C66	0.8416 (5)	0.0853 (5)	0.3276 (3)	0.0426 (17)	
H66	0.8207	0.0234	0.3256	0.051*	
C67	0.6337 (5)	0.0041 (4)	0.2601 (3)	0.0305 (13)	
C68	0.7066 (6)	−0.0709 (4)	0.2393 (3)	0.0425 (16)	
H68	0.7694	−0.0627	0.2056	0.051*	
C69	0.6860 (8)	−0.1578 (4)	0.2685 (4)	0.060 (2)	
H69	0.7362	−0.2087	0.2553	0.072*	
C70	0.5940 (7)	−0.1710 (5)	0.3161 (4)	0.057 (2)	
H70	0.5815	−0.2305	0.3359	0.068*	
C71	0.5210 (6)	−0.0990 (5)	0.3348 (4)	0.0523 (19)	
H71	0.4565	−0.1087	0.3668	0.063*	
C72	0.5402 (5)	−0.0107 (4)	0.3072 (3)	0.0393 (15)	
H72	0.4889	0.0394	0.3208	0.047*	
F1	1.0254 (7)	0.8237 (6)	0.4465 (4)	0.080 (3)	0.50
F2	0.8632 (5)	0.8126 (5)	0.4040 (4)	0.056 (2)	0.50
F3	0.9807 (6)	0.9013 (5)	0.3532 (4)	0.053 (2)	0.50
O1	1.0064 (8)	0.7603 (7)	0.2714 (5)	0.054 (3)	0.50
O2	1.0643 (10)	0.6786 (7)	0.3636 (6)	0.048 (3)	0.50
O3	1.0074 (10)	0.6640 (8)	0.1419 (7)	0.084 (4)	0.50
H3	1.0100	0.6902	0.1816	0.127*	0.50
C73	1.0177 (9)	0.7479 (7)	0.3359 (5)	0.045 (3)	0.50
C74	0.9723 (7)	0.8223 (6)	0.3867 (4)	0.030 (3)	0.50
C75	0.934 (2)	0.7209 (18)	0.1002 (14)	0.114 (9)	0.50
H75A	0.8819	0.7583	0.1327	0.171*	0.50
H75B	0.8904	0.6820	0.0713	0.171*	0.50
H75C	0.9784	0.7614	0.0681	0.171*	0.50
F1'	1.0116 (10)	0.7034 (9)	0.0846 (9)	0.152 (6)	0.50
F2'	0.9035 (8)	0.8315 (6)	0.0905 (5)	0.090 (3)	0.50
F3'	0.8372 (8)	0.7000 (7)	0.1304 (5)	0.090 (3)	0.50
O1'	1.0273 (8)	0.7022 (7)	0.2278 (7)	0.068 (3)	0.50
O2'	0.9423 (8)	0.8392 (6)	0.2283 (6)	0.071 (3)	0.50
O1W'	1.056 (2)	0.7100 (17)	0.3790 (13)	0.167 (11)	0.50
H1W1	1.0467	0.7070	0.3346	0.250*	0.50
H2W2	1.0892	0.7577	0.3869	0.250*	0.50
C73'	0.9718 (9)	0.7654 (7)	0.1983 (6)	0.046 (3)	0.50
C74'	0.9304 (9)	0.7507 (7)	0.1247 (6)	0.058 (5)	0.50

### Atomic displacement parameters (Å^2^)

**Table:**

	*U*^11^	*U*^22^	*U*^33^	*U*^12^	*U*^13^	*U*^23^
Ag1	0.02018 (18)	0.0189 (2)	0.01837 (19)	−0.00101 (15)	−0.00220 (14)	−0.00194 (14)
As1	0.0250 (3)	0.0170 (3)	0.0210 (3)	−0.0026 (2)	−0.0035 (2)	−0.0024 (2)
As2	0.0182 (2)	0.0200 (3)	0.0202 (3)	−0.0031 (2)	−0.0033 (2)	−0.0019 (2)
As3	0.0369 (3)	0.0274 (3)	0.0162 (3)	0.0014 (2)	0.0018 (2)	0.0005 (2)
As4	0.0208 (3)	0.0198 (3)	0.0250 (3)	0.0025 (2)	−0.0044 (2)	−0.0053 (2)
C1	0.027 (3)	0.025 (4)	0.028 (3)	0.002 (3)	−0.002 (2)	−0.005 (2)
C2	0.014 (5)	0.033 (4)	0.028 (5)	−0.001 (3)	−0.011 (4)	−0.012 (3)
C3	0.037 (4)	0.060 (6)	0.050 (5)	−0.003 (4)	0.005 (4)	−0.023 (5)
C4	0.061 (6)	0.061 (8)	0.030 (4)	−0.005 (6)	0.005 (4)	−0.018 (5)
C5	0.072 (7)	0.076 (7)	0.036 (5)	0.003 (5)	0.008 (5)	0.018 (5)
C6	0.049 (5)	0.033 (6)	0.025 (4)	0.000 (4)	0.002 (4)	0.000 (4)
C1'	0.027 (3)	0.025 (4)	0.028 (3)	0.002 (3)	−0.002 (2)	−0.005 (2)
C2'	0.014 (5)	0.033 (4)	0.028 (5)	−0.001 (3)	−0.011 (4)	−0.012 (3)
C3'	0.037 (4)	0.060 (6)	0.050 (5)	−0.003 (4)	0.005 (4)	−0.023 (5)
C4'	0.061 (6)	0.061 (8)	0.030 (4)	−0.005 (6)	0.005 (4)	−0.018 (5)
C5'	0.072 (7)	0.076 (7)	0.036 (5)	0.003 (5)	0.008 (5)	0.018 (5)
C6'	0.049 (5)	0.033 (6)	0.025 (4)	0.000 (4)	0.002 (4)	0.000 (4)
C7	0.027 (3)	0.018 (3)	0.020 (3)	0.002 (2)	−0.003 (2)	−0.003 (2)
C8	0.039 (3)	0.023 (3)	0.022 (3)	0.001 (2)	−0.004 (2)	−0.001 (2)
C9	0.059 (4)	0.034 (3)	0.030 (3)	0.012 (3)	−0.014 (3)	−0.007 (3)
C10	0.038 (3)	0.032 (3)	0.050 (4)	0.007 (3)	−0.022 (3)	−0.023 (3)
C11	0.026 (3)	0.036 (4)	0.055 (4)	−0.003 (3)	−0.005 (3)	−0.020 (3)
C12	0.026 (3)	0.024 (3)	0.038 (3)	−0.006 (2)	0.002 (2)	−0.006 (2)
C13	0.028 (3)	0.020 (3)	0.022 (3)	−0.003 (2)	−0.003 (2)	0.003 (2)
C14	0.028 (3)	0.023 (3)	0.038 (3)	−0.005 (2)	−0.005 (2)	0.001 (2)
C15	0.040 (3)	0.022 (3)	0.040 (3)	0.000 (3)	−0.006 (3)	−0.007 (3)
C16	0.039 (3)	0.030 (3)	0.031 (3)	0.011 (3)	−0.005 (3)	−0.001 (3)
C17	0.047 (4)	0.042 (4)	0.081 (5)	0.009 (3)	−0.036 (4)	−0.014 (4)
C18	0.043 (4)	0.028 (3)	0.077 (5)	0.008 (3)	−0.031 (4)	−0.021 (3)
C19	0.019 (2)	0.020 (2)	0.022 (3)	−0.008 (2)	0.000 (2)	0.000 (2)
C20	0.029 (5)	0.030 (7)	0.038 (4)	0.001 (4)	−0.003 (4)	−0.004 (4)
C21	0.037 (5)	0.034 (6)	0.036 (5)	0.003 (4)	0.007 (4)	−0.005 (4)
C22	0.025 (3)	0.036 (4)	0.047 (4)	0.007 (3)	0.001 (3)	−0.004 (3)
C23	0.028 (5)	0.038 (6)	0.040 (4)	0.006 (4)	−0.008 (4)	−0.006 (5)
C24	0.026 (5)	0.026 (6)	0.034 (4)	0.002 (4)	−0.006 (3)	−0.005 (5)
C19'	0.019 (2)	0.020 (2)	0.022 (3)	−0.008 (2)	0.000 (2)	0.000 (2)
C20'	0.029 (5)	0.030 (7)	0.038 (4)	0.001 (4)	−0.003 (4)	−0.004 (4)
C21'	0.037 (5)	0.034 (6)	0.036 (5)	0.003 (4)	0.007 (4)	−0.005 (4)
C22'	0.025 (3)	0.036 (4)	0.047 (4)	0.007 (3)	0.001 (3)	−0.004 (3)
C23'	0.028 (5)	0.038 (6)	0.040 (4)	0.006 (4)	−0.008 (4)	−0.006 (5)
C24'	0.026 (5)	0.026 (6)	0.034 (4)	0.002 (4)	−0.006 (3)	−0.005 (5)
C25	0.024 (3)	0.025 (3)	0.018 (3)	0.001 (2)	−0.005 (2)	−0.001 (2)
C26	0.021 (3)	0.030 (3)	0.033 (3)	0.001 (2)	0.003 (2)	0.007 (2)
C27	0.037 (3)	0.022 (3)	0.048 (4)	0.005 (3)	0.005 (3)	0.006 (3)
C28	0.042 (3)	0.021 (3)	0.036 (3)	−0.005 (3)	−0.001 (3)	0.003 (2)
C29	0.030 (3)	0.032 (3)	0.051 (4)	−0.009 (3)	−0.010 (3)	0.008 (3)
C30	0.021 (3)	0.025 (3)	0.040 (3)	−0.005 (2)	−0.004 (2)	0.008 (2)
C31	0.019 (2)	0.019 (3)	0.024 (3)	0.002 (2)	−0.004 (2)	0.001 (2)
C32	0.042 (3)	0.028 (3)	0.022 (3)	0.001 (3)	0.000 (2)	0.001 (2)
C33	0.071 (5)	0.033 (3)	0.022 (3)	0.005 (3)	−0.001 (3)	−0.012 (3)
C34	0.062 (4)	0.038 (4)	0.018 (3)	0.014 (3)	0.013 (3)	0.003 (3)
C35	0.034 (3)	0.042 (4)	0.036 (3)	0.004 (3)	0.010 (3)	0.014 (3)
C36	0.029 (3)	0.030 (3)	0.034 (3)	−0.004 (2)	−0.001 (2)	0.005 (3)
C37	0.039 (3)	0.038 (3)	0.025 (3)	−0.001 (3)	−0.001 (2)	0.002 (3)
C38	0.034 (3)	0.043 (4)	0.033 (3)	−0.003 (3)	−0.003 (3)	0.001 (3)
C39	0.052 (4)	0.046 (4)	0.048 (4)	−0.016 (3)	−0.012 (3)	−0.003 (3)
C40	0.087 (6)	0.061 (5)	0.074 (6)	−0.036 (5)	−0.029 (5)	0.037 (4)
C41	0.119 (7)	0.067 (6)	0.068 (6)	−0.044 (5)	−0.050 (5)	0.039 (5)
C42	0.075 (5)	0.056 (5)	0.040 (4)	−0.034 (4)	−0.024 (4)	0.025 (3)
C43	0.036 (3)	0.030 (3)	0.018 (3)	−0.001 (2)	0.006 (2)	0.004 (2)
C44	0.049 (4)	0.046 (4)	0.037 (4)	0.012 (3)	−0.005 (3)	−0.009 (3)
C45	0.048 (4)	0.054 (4)	0.039 (4)	0.009 (3)	−0.006 (3)	0.000 (3)
C46	0.040 (4)	0.046 (4)	0.049 (4)	−0.010 (3)	−0.011 (3)	0.004 (3)
C47	0.049 (4)	0.060 (5)	0.048 (4)	0.004 (4)	−0.010 (3)	−0.028 (4)
C48	0.039 (3)	0.050 (4)	0.032 (3)	0.005 (3)	−0.003 (3)	−0.009 (3)
C49	0.038 (3)	0.038 (3)	0.015 (3)	0.001 (3)	−0.003 (2)	−0.005 (2)
C50	0.041 (3)	0.048 (4)	0.023 (3)	0.003 (3)	−0.006 (3)	−0.013 (3)
C51	0.033 (3)	0.071 (5)	0.039 (4)	0.002 (3)	−0.002 (3)	−0.024 (4)
C52	0.047 (4)	0.072 (5)	0.034 (4)	0.026 (4)	−0.018 (3)	−0.036 (4)
C53	0.072 (5)	0.043 (4)	0.032 (3)	0.018 (4)	−0.018 (3)	−0.012 (3)
C54	0.059 (4)	0.040 (4)	0.020 (3)	0.014 (3)	−0.001 (3)	−0.006 (3)
C55	0.023 (3)	0.023 (3)	0.029 (3)	0.008 (2)	−0.004 (2)	−0.006 (2)
C56	0.031 (3)	0.026 (3)	0.035 (3)	0.001 (2)	−0.009 (2)	−0.006 (2)
C57	0.051 (4)	0.034 (3)	0.033 (3)	0.005 (3)	−0.011 (3)	−0.012 (3)
C58	0.053 (4)	0.048 (4)	0.030 (3)	0.016 (3)	0.003 (3)	−0.012 (3)
C59	0.030 (3)	0.072 (5)	0.041 (4)	0.016 (3)	0.004 (3)	−0.015 (4)
C60	0.028 (3)	0.057 (4)	0.035 (3)	0.008 (3)	−0.005 (3)	−0.010 (3)
C61	0.020 (3)	0.030 (3)	0.025 (3)	0.001 (2)	0.001 (2)	−0.006 (2)
C62	0.043 (4)	0.034 (4)	0.089 (6)	0.007 (3)	−0.034 (4)	−0.018 (4)
C63	0.047 (4)	0.050 (5)	0.100 (6)	0.000 (4)	−0.033 (4)	−0.038 (4)
C64	0.025 (3)	0.075 (5)	0.042 (4)	−0.012 (3)	−0.008 (3)	−0.018 (4)
C65	0.044 (4)	0.073 (5)	0.061 (5)	−0.016 (4)	−0.030 (4)	0.021 (4)
C66	0.033 (3)	0.048 (4)	0.051 (4)	−0.016 (3)	−0.021 (3)	0.018 (3)
C67	0.037 (3)	0.033 (3)	0.024 (3)	−0.006 (3)	−0.011 (2)	−0.005 (2)
C68	0.072 (5)	0.026 (3)	0.029 (3)	0.010 (3)	−0.008 (3)	−0.004 (3)
C69	0.119 (7)	0.025 (3)	0.036 (4)	0.013 (4)	−0.024 (4)	−0.007 (3)
C70	0.107 (7)	0.029 (4)	0.040 (4)	−0.024 (4)	−0.029 (4)	0.006 (3)
C71	0.065 (5)	0.046 (4)	0.050 (4)	−0.026 (4)	−0.019 (4)	0.009 (3)
C72	0.037 (3)	0.031 (3)	0.052 (4)	−0.007 (3)	−0.014 (3)	0.001 (3)
F1	0.090 (6)	0.083 (6)	0.069 (5)	0.002 (5)	−0.024 (5)	−0.031 (5)
F2	0.046 (4)	0.081 (5)	0.037 (4)	0.019 (4)	0.014 (3)	0.000 (4)
F3	0.047 (4)	0.049 (4)	0.062 (5)	−0.003 (3)	0.014 (4)	−0.011 (4)
O1	0.047 (5)	0.069 (6)	0.049 (5)	−0.033 (5)	0.001 (4)	−0.011 (5)
O2	0.050 (5)	0.034 (5)	0.057 (6)	0.025 (4)	−0.002 (4)	0.005 (4)
O3	0.088 (7)	0.078 (7)	0.082 (7)	0.027 (6)	0.011 (6)	0.002 (6)
C73	0.030 (6)	0.052 (7)	0.056 (7)	−0.020 (5)	−0.008 (5)	0.009 (6)
C74	0.031 (5)	0.042 (6)	0.021 (5)	−0.012 (5)	−0.006 (4)	−0.007 (4)
C75	0.092 (12)	0.145 (13)	0.109 (12)	0.004 (9)	−0.026 (9)	−0.016 (9)
F1'	0.141 (9)	0.176 (10)	0.133 (9)	0.025 (8)	0.011 (8)	−0.023 (8)
F2'	0.077 (6)	0.116 (7)	0.075 (6)	−0.001 (5)	−0.005 (5)	0.038 (5)
F3'	0.101 (6)	0.102 (7)	0.075 (6)	−0.043 (6)	−0.021 (5)	0.000 (5)
O1'	0.050 (5)	0.066 (6)	0.089 (7)	0.000 (5)	−0.009 (5)	0.008 (6)
O2'	0.043 (5)	0.057 (6)	0.112 (7)	0.003 (5)	0.002 (5)	−0.002 (6)
O1W'	0.166 (13)	0.177 (14)	0.163 (14)	−0.025 (9)	−0.033 (9)	0.001 (9)
C73'	0.026 (5)	0.045 (6)	0.066 (7)	−0.010 (5)	0.002 (5)	0.005 (6)
C74'	0.050 (8)	0.078 (9)	0.044 (7)	0.009 (7)	0.002 (6)	0.008 (7)

### Geometric parameters (Å, º)

**Table:**

Ag1—As1	2.6464 (6)	C31—C36	1.393 (7)
Ag1—As2	2.6460 (6)	C32—C33	1.391 (8)
Ag1—As3	2.6342 (7)	C32—H32	0.9500
Ag1—As4	2.6494 (6)	C33—C34	1.377 (9)
As1—C1	1.917 (5)	C33—H33	0.9500
As1—C7	1.946 (5)	C34—C35	1.384 (9)
As1—C13	1.948 (5)	C34—H34	0.9500
As1—C1'	1.955 (5)	C35—C36	1.384 (8)
As2—C31	1.938 (5)	C35—H35	0.9500
As2—C19	1.947 (5)	C36—H36	0.9500
As2—C19'	1.947 (5)	C37—C38	1.379 (8)
As2—C25	1.947 (5)	C37—C42	1.386 (8)
As3—C49	1.940 (6)	C38—C39	1.373 (8)
As3—C37	1.941 (6)	C38—H38	0.9500
As3—C43	1.946 (6)	C39—C40	1.375 (9)
As4—C67	1.932 (6)	C39—H39	0.9500
As4—C55	1.944 (5)	C40—C41	1.395 (10)
As4—C61	1.952 (5)	C40—H40	0.9500
C1—C2	1.3900	C41—C42	1.364 (9)
C1—C6	1.3900	C41—H41	0.9500
C2—C3	1.3900	C42—H42	0.9500
C2—H2	0.9500	C43—C48	1.374 (8)
C3—C4	1.3900	C43—C44	1.379 (8)
C3—H3A	0.9500	C44—C45	1.400 (9)
C4—C5	1.3900	C44—H44	0.9500
C4—H4	0.9500	C45—C46	1.369 (9)
C5—C6	1.3900	C45—H45	0.9500
C5—H5	0.9500	C46—C47	1.361 (9)
C6—H6	0.9500	C46—H46	0.9500
C1'—C2'	1.3900	C47—C48	1.396 (8)
C1'—C6'	1.3900	C47—H47	0.9500
C2'—C3'	1.3900	C48—H48	0.9500
C2'—H2'	0.9500	C49—C54	1.377 (8)
C3'—C4'	1.3900	C49—C50	1.393 (8)
C3'—H3'	0.9500	C50—C51	1.380 (9)
C4'—C5'	1.3900	C50—H50	0.9500
C4'—H4'	0.9500	C51—C52	1.375 (10)
C5'—C6'	1.3900	C51—H51	0.9500
C5'—H5'	0.9500	C52—C53	1.382 (10)
C6'—H6'	0.9500	C52—H52	0.9500
C7—C8	1.383 (7)	C53—C54	1.396 (8)
C7—C12	1.385 (7)	C53—H53	0.9500
C8—C9	1.389 (8)	C54—H54	0.9500
C8—H8	0.9500	C55—C56	1.394 (7)
C9—C10	1.379 (9)	C55—C60	1.398 (7)
C9—H9	0.9500	C56—C57	1.387 (8)
C10—C11	1.376 (9)	C56—H56	0.9500
C10—H10	0.9500	C57—C58	1.369 (8)
C11—C12	1.383 (8)	C57—H57	0.9500
C11—H11	0.9500	C58—C59	1.395 (9)
C12—H12	0.9500	C58—H58	0.9500
C13—C14	1.373 (7)	C59—C60	1.373 (8)
C13—C18	1.380 (7)	C59—H59	0.9500
C14—C15	1.389 (7)	C60—H60	0.9500
C14—H14	0.9500	C61—C62	1.369 (8)
C15—C16	1.368 (8)	C61—C66	1.369 (7)
C15—H15	0.9500	C62—C63	1.392 (8)
C16—C17	1.369 (9)	C62—H62	0.9500
C16—H16	0.9500	C63—C64	1.334 (10)
C17—C18	1.389 (8)	C63—H63	0.9500
C17—H17	0.9500	C64—C65	1.345 (10)
C18—H18	0.9500	C64—H64	0.9500
C19—C20	1.3900	C65—C66	1.396 (8)
C19—C24	1.3900	C65—H65	0.9500
C20—C21	1.3900	C66—H66	0.9500
C20—H20	0.9500	C67—C72	1.388 (8)
C21—C22	1.3900	C67—C68	1.398 (8)
C21—H21	0.9500	C68—C69	1.394 (9)
C22—C23	1.3900	C68—H68	0.9500
C22—H22	0.9500	C69—C70	1.376 (11)
C23—C24	1.3900	C69—H69	0.9500
C23—H23	0.9500	C70—C71	1.357 (10)
C24—H24	0.9500	C70—H70	0.9500
C19'—C20'	1.3900	C71—C72	1.399 (8)
C19'—C24'	1.3900	C71—H71	0.9500
C20'—C21'	1.3900	C72—H72	0.9500
C20'—H20B	0.9500	F1—C74	1.318 (8)
C21'—C22'	1.3900	F2—C74	1.330 (8)
C21'—H21B	0.9500	F3—C74	1.300 (8)
C22'—C23'	1.3900	O1—C73	1.223 (8)
C22'—H22B	0.9500	O2—C73	1.244 (8)
C23'—C24'	1.3900	O3—C75	1.431 (10)
C23'—H23B	0.9500	O3—H3	0.8400
C24'—H24B	0.9500	C73—C74	1.501 (9)
C25—C30	1.384 (7)	C75—H75A	0.9800
C25—C26	1.388 (7)	C75—H75B	0.9800
C26—C27	1.388 (7)	C75—H75C	0.9800
C26—H26	0.9500	F1'—C74'	1.342 (9)
C27—C28	1.385 (8)	F2'—C74'	1.359 (9)
C27—H27	0.9500	F3'—C74'	1.363 (9)
C28—C29	1.372 (8)	O1'—C73'	1.244 (8)
C28—H28	0.9500	O2'—C73'	1.241 (8)
C29—C30	1.387 (7)	O1W'—H1W1	0.8401
C29—H29	0.9500	O1W'—H2W2	0.8400
C30—H30	0.9500	C73'—C74'	1.509 (9)
C31—C32	1.392 (7)		
			
As3—Ag1—As2	109.89 (2)	C29—C28—H28	120.4
As3—Ag1—As1	109.00 (2)	C27—C28—H28	120.4
As2—Ag1—As1	110.713 (19)	C28—C29—C30	121.4 (5)
As3—Ag1—As4	108.64 (2)	C28—C29—H29	119.3
As2—Ag1—As4	108.65 (2)	C30—C29—H29	119.3
As1—Ag1—As4	109.92 (2)	C25—C30—C29	119.3 (5)
C1—As1—C7	102.1 (3)	C25—C30—H30	120.4
C1—As1—C13	105.3 (3)	C29—C30—H30	120.4
C7—As1—C13	99.5 (2)	C32—C31—C36	119.3 (5)
C7—As1—C1'	100.6 (3)	C32—C31—As2	121.8 (4)
C13—As1—C1'	99.9 (4)	C36—C31—As2	118.8 (4)
C1—As1—Ag1	110.8 (3)	C33—C32—C31	120.1 (5)
C7—As1—Ag1	119.45 (14)	C33—C32—H32	119.9
C13—As1—Ag1	117.58 (15)	C31—C32—H32	119.9
C1'—As1—Ag1	116.3 (3)	C34—C33—C32	120.0 (6)
C31—As2—C19	100.8 (4)	C34—C33—H33	120.0
C31—As2—C19'	100.5 (4)	C32—C33—H33	120.0
C31—As2—C25	101.8 (2)	C33—C34—C35	120.3 (5)
C19—As2—C25	100.1 (3)	C33—C34—H34	119.9
C19'—As2—C25	101.3 (3)	C35—C34—H34	119.9
C31—As2—Ag1	115.77 (14)	C34—C35—C36	120.1 (6)
C19—As2—Ag1	120.7 (4)	C34—C35—H35	119.9
C19'—As2—Ag1	120.0 (4)	C36—C35—H35	119.9
C25—As2—Ag1	114.70 (14)	C35—C36—C31	120.1 (6)
C49—As3—C37	100.6 (2)	C35—C36—H36	119.9
C49—As3—C43	102.0 (2)	C31—C36—H36	119.9
C37—As3—C43	102.4 (2)	C38—C37—C42	120.4 (6)
C49—As3—Ag1	113.53 (15)	C38—C37—As3	118.1 (4)
C37—As3—Ag1	118.41 (18)	C42—C37—As3	121.5 (5)
C43—As3—Ag1	117.25 (15)	C39—C38—C37	120.3 (5)
C67—As4—C55	102.0 (2)	C39—C38—H38	119.9
C67—As4—C61	100.7 (2)	C37—C38—H38	119.9
C55—As4—C61	101.4 (2)	C38—C39—C40	120.2 (6)
C67—As4—Ag1	118.22 (18)	C38—C39—H39	119.9
C55—As4—Ag1	119.10 (14)	C40—C39—H39	119.9
C61—As4—Ag1	112.61 (16)	C39—C40—C41	118.8 (7)
C2—C1—C6	120.0	C39—C40—H40	120.6
C2—C1—As1	117.7 (6)	C41—C40—H40	120.6
C6—C1—As1	122.2 (6)	C42—C41—C40	121.6 (7)
C1—C2—C3	120.0	C42—C41—H41	119.2
C1—C2—H2	120.0	C40—C41—H41	119.2
C3—C2—H2	120.0	C41—C42—C37	118.6 (6)
C4—C3—C2	120.0	C41—C42—H42	120.7
C4—C3—H3A	120.0	C37—C42—H42	120.7
C2—C3—H3A	120.0	C48—C43—C44	119.6 (6)
C5—C4—C3	120.0	C48—C43—As3	122.0 (4)
C5—C4—H4	120.0	C44—C43—As3	118.3 (4)
C3—C4—H4	120.0	C43—C44—C45	119.3 (6)
C4—C5—C6	120.0	C43—C44—H44	120.3
C4—C5—H5	120.0	C45—C44—H44	120.3
C6—C5—H5	120.0	C46—C45—C44	120.8 (6)
C5—C6—C1	120.0	C46—C45—H45	119.6
C5—C6—H6	120.0	C44—C45—H45	119.6
C1—C6—H6	120.0	C47—C46—C45	119.6 (6)
C2'—C1'—C6'	120.0	C47—C46—H46	120.2
C2'—C1'—As1	117.0 (6)	C45—C46—H46	120.2
C6'—C1'—As1	122.5 (6)	C46—C47—C48	120.4 (6)
C1'—C2'—C3'	120.0	C46—C47—H47	119.8
C1'—C2'—H2'	120.0	C48—C47—H47	119.8
C3'—C2'—H2'	120.0	C43—C48—C47	120.2 (6)
C2'—C3'—C4'	120.0	C43—C48—H48	119.9
C2'—C3'—H3'	120.0	C47—C48—H48	119.9
C4'—C3'—H3'	120.0	C54—C49—C50	120.2 (6)
C5'—C4'—C3'	120.0	C54—C49—As3	118.3 (4)
C5'—C4'—H4'	120.0	C50—C49—As3	121.4 (4)
C3'—C4'—H4'	120.0	C51—C50—C49	119.8 (6)
C4'—C5'—C6'	120.0	C51—C50—H50	120.1
C4'—C5'—H5'	120.0	C49—C50—H50	120.1
C6'—C5'—H5'	120.0	C52—C51—C50	120.1 (6)
C5'—C6'—C1'	120.0	C52—C51—H51	119.9
C5'—C6'—H6'	120.0	C50—C51—H51	119.9
C1'—C6'—H6'	120.0	C51—C52—C53	120.4 (6)
C8—C7—C12	119.3 (5)	C51—C52—H52	119.8
C8—C7—As1	118.7 (4)	C53—C52—H52	119.8
C12—C7—As1	121.8 (4)	C52—C53—C54	119.8 (6)
C7—C8—C9	120.0 (5)	C52—C53—H53	120.1
C7—C8—H8	120.0	C54—C53—H53	120.1
C9—C8—H8	120.0	C49—C54—C53	119.5 (6)
C10—C9—C8	120.2 (6)	C49—C54—H54	120.2
C10—C9—H9	119.9	C53—C54—H54	120.2
C8—C9—H9	119.9	C56—C55—C60	118.5 (5)
C11—C10—C9	119.9 (5)	C56—C55—As4	118.4 (4)
C11—C10—H10	120.1	C60—C55—As4	123.0 (4)
C9—C10—H10	120.1	C57—C56—C55	120.2 (5)
C10—C11—C12	120.0 (6)	C57—C56—H56	119.9
C10—C11—H11	120.0	C55—C56—H56	119.9
C12—C11—H11	120.0	C58—C57—C56	120.8 (6)
C11—C12—C7	120.5 (5)	C58—C57—H57	119.6
C11—C12—H12	119.8	C56—C57—H57	119.6
C7—C12—H12	119.8	C57—C58—C59	119.5 (6)
C14—C13—C18	119.0 (5)	C57—C58—H58	120.3
C14—C13—As1	122.5 (4)	C59—C58—H58	120.3
C18—C13—As1	118.4 (4)	C60—C59—C58	120.2 (6)
C13—C14—C15	120.5 (5)	C60—C59—H59	119.9
C13—C14—H14	119.8	C58—C59—H59	119.9
C15—C14—H14	119.8	C59—C60—C55	120.7 (5)
C16—C15—C14	120.4 (5)	C59—C60—H60	119.6
C16—C15—H15	119.8	C55—C60—H60	119.6
C14—C15—H15	119.8	C62—C61—C66	118.9 (5)
C15—C16—C17	119.5 (5)	C62—C61—As4	118.4 (4)
C15—C16—H16	120.3	C66—C61—As4	122.7 (4)
C17—C16—H16	120.3	C61—C62—C63	119.9 (6)
C16—C17—C18	120.4 (6)	C61—C62—H62	120.0
C16—C17—H17	119.8	C63—C62—H62	120.0
C18—C17—H17	119.8	C64—C63—C62	120.8 (7)
C13—C18—C17	120.2 (6)	C64—C63—H63	119.6
C13—C18—H18	119.9	C62—C63—H63	119.6
C17—C18—H18	119.9	C63—C64—C65	120.0 (6)
C20—C19—C24	120.0	C63—C64—H64	120.0
C20—C19—As2	120.1 (7)	C65—C64—H64	120.0
C24—C19—As2	119.9 (7)	C64—C65—C66	120.7 (6)
C19—C20—C21	120.0	C64—C65—H65	119.7
C19—C20—H20	120.0	C66—C65—H65	119.7
C21—C20—H20	120.0	C61—C66—C65	119.6 (6)
C22—C21—C20	120.0	C61—C66—H66	120.2
C22—C21—H21	120.0	C65—C66—H66	120.2
C20—C21—H21	120.0	C72—C67—C68	119.0 (6)
C23—C22—C21	120.0	C72—C67—As4	118.3 (5)
C23—C22—H22	120.0	C68—C67—As4	122.7 (5)
C21—C22—H22	120.0	C69—C68—C67	119.3 (7)
C24—C23—C22	120.0	C69—C68—H68	120.4
C24—C23—H23	120.0	C67—C68—H68	120.4
C22—C23—H23	120.0	C70—C69—C68	121.0 (7)
C23—C24—C19	120.0	C70—C69—H69	119.5
C23—C24—H24	120.0	C68—C69—H69	119.5
C19—C24—H24	120.0	C71—C70—C69	120.1 (6)
C20'—C19'—C24'	120.0	C71—C70—H70	120.0
C20'—C19'—As2	117.7 (7)	C69—C70—H70	120.0
C24'—C19'—As2	122.2 (7)	C70—C71—C72	120.3 (7)
C21'—C20'—C19'	120.0	C70—C71—H71	119.9
C21'—C20'—H20B	120.0	C72—C71—H71	119.9
C19'—C20'—H20B	120.0	C67—C72—C71	120.4 (6)
C20'—C21'—C22'	120.0	C67—C72—H72	119.8
C20'—C21'—H21B	120.0	C71—C72—H72	119.8
C22'—C21'—H21B	120.0	C75—O3—H3	106.6
C21'—C22'—C23'	120.0	O1—C73—O2	125.7 (10)
C21'—C22'—H22B	120.0	O1—C73—C74	117.8 (9)
C23'—C22'—H22B	120.0	O2—C73—C74	116.5 (9)
C24'—C23'—C22'	120.0	F3—C74—F1	109.0 (7)
C24'—C23'—H23B	120.0	F3—C74—F2	107.1 (7)
C22'—C23'—H23B	120.0	F1—C74—F2	108.9 (7)
C23'—C24'—C19'	120.0	F3—C74—C73	108.4 (7)
C23'—C24'—H24B	120.0	F1—C74—C73	113.4 (8)
C19'—C24'—H24B	120.0	F2—C74—C73	109.9 (8)
C30—C25—C26	119.9 (5)	H1W1—O1W'—H2W2	109.5
C30—C25—As2	122.4 (4)	O2'—C73'—O1'	123.2 (10)
C26—C25—As2	117.7 (4)	O2'—C73'—C74'	117.2 (10)
C27—C26—C25	120.0 (5)	O1'—C73'—C74'	119.5 (10)
C27—C26—H26	120.0	F1'—C74'—F2'	109.5 (8)
C25—C26—H26	120.0	F1'—C74'—F3'	107.9 (9)
C28—C27—C26	120.3 (5)	F2'—C74'—F3'	108.2 (8)
C28—C27—H27	119.9	F1'—C74'—C73'	108.6 (11)
C26—C27—H27	119.9	F2'—C74'—C73'	112.2 (9)
C29—C28—C27	119.2 (5)	F3'—C74'—C73'	110.5 (9)
			
As3—Ag1—As1—C1	172.1 (3)	C20'—C21'—C22'—C23'	0.0
As2—Ag1—As1—C1	−67.0 (3)	C21'—C22'—C23'—C24'	0.0
As4—Ag1—As1—C1	53.1 (3)	C22'—C23'—C24'—C19'	0.0
As3—Ag1—As1—C7	53.82 (17)	C20'—C19'—C24'—C23'	0.0
As2—Ag1—As1—C7	174.81 (17)	As2—C19'—C24'—C23'	−176.8 (8)
As4—Ag1—As1—C7	−65.15 (17)	C31—As2—C25—C30	−113.0 (4)
As3—Ag1—As1—C13	−66.81 (17)	C19—As2—C25—C30	−9.6 (6)
As2—Ag1—As1—C13	54.18 (17)	C19'—As2—C25—C30	−9.6 (6)
As4—Ag1—As1—C13	174.22 (17)	Ag1—As2—C25—C30	121.2 (4)
As3—Ag1—As1—C1'	174.8 (3)	C31—As2—C25—C26	69.3 (4)
As2—Ag1—As1—C1'	−64.2 (3)	C19—As2—C25—C26	172.8 (6)
As4—Ag1—As1—C1'	55.9 (3)	C19'—As2—C25—C26	172.7 (5)
As3—Ag1—As2—C31	−175.46 (16)	Ag1—As2—C25—C26	−56.5 (4)
As1—Ag1—As2—C31	64.09 (16)	C30—C25—C26—C27	0.7 (8)
As4—Ag1—As2—C31	−56.72 (16)	As2—C25—C26—C27	178.4 (4)
As3—Ag1—As2—C19	62.6 (4)	C25—C26—C27—C28	−0.5 (9)
As1—Ag1—As2—C19	−57.9 (4)	C26—C27—C28—C29	0.7 (9)
As4—Ag1—As2—C19	−178.7 (4)	C27—C28—C29—C30	−1.1 (9)
As3—Ag1—As2—C19'	63.7 (4)	C26—C25—C30—C29	−1.0 (8)
As1—Ag1—As2—C19'	−56.7 (4)	As2—C25—C30—C29	−178.6 (4)
As4—Ag1—As2—C19'	−177.6 (4)	C28—C29—C30—C25	1.2 (9)
As3—Ag1—As2—C25	−57.31 (17)	C19—As2—C31—C32	−82.9 (5)
As1—Ag1—As2—C25	−177.77 (17)	C19'—As2—C31—C32	−84.1 (5)
As4—Ag1—As2—C25	61.42 (17)	C25—As2—C31—C32	20.0 (4)
As2—Ag1—As3—C49	−60.46 (19)	Ag1—As2—C31—C32	145.1 (4)
As1—Ag1—As3—C49	61.03 (19)	C19—As2—C31—C36	94.1 (5)
As4—Ag1—As3—C49	−179.20 (19)	C19'—As2—C31—C36	92.9 (5)
As2—Ag1—As3—C37	57.22 (19)	C25—As2—C31—C36	−163.0 (4)
As1—Ag1—As3—C37	178.71 (19)	Ag1—As2—C31—C36	−37.9 (4)
As4—Ag1—As3—C37	−61.53 (19)	C36—C31—C32—C33	−0.8 (8)
As2—Ag1—As3—C43	−179.14 (18)	As2—C31—C32—C33	176.1 (4)
As1—Ag1—As3—C43	−57.65 (18)	C31—C32—C33—C34	−0.2 (9)
As4—Ag1—As3—C43	62.12 (18)	C32—C33—C34—C35	0.9 (9)
As3—Ag1—As4—C67	53.81 (18)	C33—C34—C35—C36	−0.5 (9)
As2—Ag1—As4—C67	−65.71 (18)	C34—C35—C36—C31	−0.6 (8)
As1—Ag1—As4—C67	172.99 (18)	C32—C31—C36—C35	1.3 (8)
As3—Ag1—As4—C55	178.48 (18)	As2—C31—C36—C35	−175.8 (4)
As2—Ag1—As4—C55	58.96 (18)	C49—As3—C37—C38	77.2 (5)
As1—Ag1—As4—C55	−62.33 (18)	C43—As3—C37—C38	−177.9 (5)
As3—Ag1—As4—C61	−63.11 (17)	Ag1—As3—C37—C38	−47.1 (5)
As2—Ag1—As4—C61	177.37 (17)	C49—As3—C37—C42	−102.5 (6)
As1—Ag1—As4—C61	56.08 (17)	C43—As3—C37—C42	2.5 (6)
C7—As1—C1—C2	78.7 (5)	Ag1—As3—C37—C42	133.2 (5)
C13—As1—C1—C2	−177.7 (4)	C42—C37—C38—C39	1.4 (10)
C1'—As1—C1—C2	155 (5)	As3—C37—C38—C39	−178.2 (5)
Ag1—As1—C1—C2	−49.6 (5)	C37—C38—C39—C40	−1.0 (11)
C7—As1—C1—C6	−104.6 (6)	C38—C39—C40—C41	0.9 (13)
C13—As1—C1—C6	−1.1 (6)	C39—C40—C41—C42	−1.4 (15)
C1'—As1—C1—C6	−28 (5)	C40—C41—C42—C37	1.9 (14)
Ag1—As1—C1—C6	127.1 (5)	C38—C37—C42—C41	−1.8 (11)
C6—C1—C2—C3	0.0	As3—C37—C42—C41	177.8 (7)
As1—C1—C2—C3	176.7 (7)	C49—As3—C43—C48	6.9 (5)
C1—C2—C3—C4	0.0	C37—As3—C43—C48	−97.0 (5)
C2—C3—C4—C5	0.0	Ag1—As3—C43—C48	131.5 (4)
C3—C4—C5—C6	0.0	C49—As3—C43—C44	−169.8 (4)
C4—C5—C6—C1	0.0	C37—As3—C43—C44	86.3 (5)
C2—C1—C6—C5	0.0	Ag1—As3—C43—C44	−45.2 (5)
As1—C1—C6—C5	−176.6 (7)	C48—C43—C44—C45	0.9 (9)
C1—As1—C1'—C2'	−5 (5)	As3—C43—C44—C45	177.7 (5)
C7—As1—C1'—C2'	100.1 (5)	C43—C44—C45—C46	0.1 (10)
C13—As1—C1'—C2'	−158.1 (4)	C44—C45—C46—C47	−1.2 (10)
Ag1—As1—C1'—C2'	−30.4 (5)	C45—C46—C47—C48	1.3 (10)
C1—As1—C1'—C6'	−177 (5)	C44—C43—C48—C47	−0.9 (9)
C7—As1—C1'—C6'	−71.8 (5)	As3—C43—C48—C47	−177.5 (5)
C13—As1—C1'—C6'	30.0 (5)	C46—C47—C48—C43	−0.2 (10)
Ag1—As1—C1'—C6'	157.6 (4)	C37—As3—C49—C54	−164.2 (4)
C6'—C1'—C2'—C3'	0.0	C43—As3—C49—C54	90.5 (5)
As1—C1'—C2'—C3'	−172.2 (7)	Ag1—As3—C49—C54	−36.7 (5)
C1'—C2'—C3'—C4'	0.0	C37—As3—C49—C50	12.7 (5)
C2'—C3'—C4'—C5'	0.0	C43—As3—C49—C50	−92.6 (5)
C3'—C4'—C5'—C6'	0.0	Ag1—As3—C49—C50	140.3 (4)
C4'—C5'—C6'—C1'	0.0	C54—C49—C50—C51	0.6 (8)
C2'—C1'—C6'—C5'	0.0	As3—C49—C50—C51	−176.3 (4)
As1—C1'—C6'—C5'	171.7 (7)	C49—C50—C51—C52	0.1 (9)
C1—As1—C7—C8	−160.0 (5)	C50—C51—C52—C53	−0.4 (9)
C13—As1—C7—C8	91.9 (4)	C51—C52—C53—C54	0.0 (9)
C1'—As1—C7—C8	−166.0 (5)	C50—C49—C54—C53	−0.9 (9)
Ag1—As1—C7—C8	−37.4 (5)	As3—C49—C54—C53	176.0 (4)
C1—As1—C7—C12	23.9 (5)	C52—C53—C54—C49	0.6 (9)
C13—As1—C7—C12	−84.1 (4)	C67—As4—C55—C56	82.8 (4)
C1'—As1—C7—C12	18.0 (5)	C61—As4—C55—C56	−173.5 (4)
Ag1—As1—C7—C12	146.5 (4)	Ag1—As4—C55—C56	−49.4 (5)
C12—C7—C8—C9	1.2 (8)	C67—As4—C55—C60	−98.3 (5)
As1—C7—C8—C9	−174.9 (4)	C61—As4—C55—C60	5.4 (5)
C7—C8—C9—C10	0.1 (8)	Ag1—As4—C55—C60	129.5 (4)
C8—C9—C10—C11	−1.8 (9)	C60—C55—C56—C57	−0.3 (8)
C9—C10—C11—C12	2.2 (9)	As4—C55—C56—C57	178.7 (4)
C10—C11—C12—C7	−0.9 (8)	C55—C56—C57—C58	1.3 (9)
C8—C7—C12—C11	−0.8 (8)	C56—C57—C58—C59	−1.4 (10)
As1—C7—C12—C11	175.2 (4)	C57—C58—C59—C60	0.4 (10)
C1—As1—C13—C14	−96.6 (5)	C58—C59—C60—C55	0.6 (10)
C7—As1—C13—C14	8.9 (5)	C56—C55—C60—C59	−0.7 (9)
C1'—As1—C13—C14	−93.8 (5)	As4—C55—C60—C59	−179.6 (5)
Ag1—As1—C13—C14	139.5 (4)	C67—As4—C61—C62	−168.3 (5)
C1—As1—C13—C18	87.1 (6)	C55—As4—C61—C62	86.9 (5)
C7—As1—C13—C18	−167.4 (5)	Ag1—As4—C61—C62	−41.4 (5)
C1'—As1—C13—C18	89.9 (6)	C67—As4—C61—C66	9.8 (5)
Ag1—As1—C13—C18	−36.9 (5)	C55—As4—C61—C66	−94.9 (5)
C18—C13—C14—C15	1.3 (9)	Ag1—As4—C61—C66	136.7 (5)
As1—C13—C14—C15	−175.0 (4)	C66—C61—C62—C63	−1.1 (10)
C13—C14—C15—C16	−0.7 (9)	As4—C61—C62—C63	177.2 (6)
C14—C15—C16—C17	0.9 (9)	C61—C62—C63—C64	2.4 (12)
C15—C16—C17—C18	−1.7 (11)	C62—C63—C64—C65	−2.5 (12)
C14—C13—C18—C17	−2.2 (10)	C63—C64—C65—C66	1.3 (12)
As1—C13—C18—C17	174.3 (6)	C62—C61—C66—C65	−0.1 (10)
C16—C17—C18—C13	2.4 (11)	As4—C61—C66—C65	−178.2 (5)
C31—As2—C19—C20	−148.8 (5)	C64—C65—C66—C61	0.0 (11)
C19'—As2—C19—C20	−75 (37)	C55—As4—C67—C72	−151.8 (4)
C25—As2—C19—C20	107.0 (5)	C61—As4—C67—C72	104.0 (4)
Ag1—As2—C19—C20	−19.9 (6)	Ag1—As4—C67—C72	−19.1 (5)
C31—As2—C19—C24	29.6 (6)	C55—As4—C67—C68	32.0 (5)
C19'—As2—C19—C24	104 (37)	C61—As4—C67—C68	−72.2 (5)
C25—As2—C19—C24	−74.6 (6)	Ag1—As4—C67—C68	164.7 (4)
Ag1—As2—C19—C24	158.5 (4)	C72—C67—C68—C69	−2.5 (9)
C24—C19—C20—C21	0.0	As4—C67—C68—C69	173.7 (5)
As2—C19—C20—C21	178.4 (8)	C67—C68—C69—C70	1.4 (10)
C19—C20—C21—C22	0.0	C68—C69—C70—C71	0.6 (10)
C20—C21—C22—C23	0.0	C69—C70—C71—C72	−1.5 (10)
C21—C22—C23—C24	0.0	C68—C67—C72—C71	1.7 (8)
C22—C23—C24—C19	0.0	As4—C67—C72—C71	−174.7 (4)
C20—C19—C24—C23	0.0	C70—C71—C72—C67	0.4 (9)
As2—C19—C24—C23	−178.4 (8)	O1—C73—C74—F3	−31.9 (13)
C31—As2—C19'—C20'	−173.9 (5)	O2—C73—C74—F3	147.7 (11)
C19—As2—C19'—C20'	80 (37)	O1—C73—C74—F1	−153.1 (10)
C25—As2—C19'—C20'	81.7 (5)	O2—C73—C74—F1	26.5 (14)
Ag1—As2—C19'—C20'	−45.7 (6)	O1—C73—C74—F2	84.7 (12)
C31—As2—C19'—C24'	2.9 (6)	O2—C73—C74—F2	−95.6 (12)
C19—As2—C19'—C24'	−103 (37)	O2'—C73'—C74'—F1'	144.6 (12)
C25—As2—C19'—C24'	−101.5 (6)	O1'—C73'—C74'—F1'	−39.7 (15)
Ag1—As2—C19'—C24'	131.1 (5)	O2'—C73'—C74'—F2'	23.5 (15)
C24'—C19'—C20'—C21'	0.0	O1'—C73'—C74'—F2'	−160.8 (11)
As2—C19'—C20'—C21'	176.9 (8)	O2'—C73'—C74'—F3'	−97.3 (13)
C19'—C20'—C21'—C22'	0.0	O1'—C73'—C74'—F3'	78.5 (14)

### Hydrogen-bond geometry (Å, º)

**Table:**

*D*—H···*A*	*D*—H	H···*A*	*D*···*A*	*D*—H···*A*
O3—H3···O1	0.84	1.97	2.80 (2)	175
O1w'—H1w1···O1′	0.84	2.02	2.86 (3)	179
